# Network-targeting combination therapy of leptomeningeal glioblastoma using multiple synthetic lethal strategies: a case report

**DOI:** 10.3389/fonc.2023.1210224

**Published:** 2023-10-31

**Authors:** Michael P. Castro, Bence Sipos, Saskia Biskup, Nina Kahn

**Affiliations:** ^1^ Personalized Cancer Medicine, PLLC, Santa Monica, CA, United States; ^2^ Beverly Hills Cancer Center, Beverly Hills, CA, United States; ^3^ Cellworks Group, Inc, San Francisco, CA, United States; ^4^ Department of Pathology, Molekularpathologie Baden-Württemberg GbR, Tuebingen, Germany; ^5^ Center for Genomics & Transcriptomics, GmbH, Tuebingen, Germany; ^6^ Independent Researcher, Amsterdam, Netherlands

**Keywords:** glioblastoma, leptomeningeal, intra-patient dose escalation, lomustine, olaparib, synthetic lethal, network-targeting combination therapy (NTCT)

## Abstract

Network targeting of disease-specific nodes represents a useful principle for designing combination cancer therapy. In this case of a patient with relapsed leptomeningeal glioblastoma, comprehensive molecular diagnosis led to the identification of a disease network characterized by multiple disease-specific synthetic lethal vulnerabilities involving DNA repair, REDOX homeostasis, and impaired autophagy which suggested a novel network-targeting combination therapy (NTCT). A treatment regimen consisting of lomustine, olaparib, digoxin, metformin, and high dose intravenous ascorbate was employed using the principle of intra-patient dose escalation to deliver the treatment with adequate safety measures to achieve a definitive clinical result.

## Background

Leptomeningeal spread of glioblastoma (LM-GBM) is a life-threatening disease renowned for dire neurological sequelae and short median survival of 1.6 to 3.8 months (1). Though responses to chemotherapy are documented and generally favor a disposition of intervention, single agents accomplish relatively little against complex diseases like LM-GBM. Remarkably, addressing this complexity may be guided by molecular diagnosis which often discloses more than one driver abnormality and/or synthetic lethal opportunity. Indeed, whole exome next generation DNA sequencing (WES) (2, 3) and detailed copy number analyses (4–6) often reveal many genomic aberrations, thereby bringing sharper focus to a particular cancer’s dysregulated signaling pathways, complex adaptive network, master regulators, and synthetic lethal vulnerabilities (7, 8).

In principle, immediately life-threatening complex cancers have a strong rationale for combination approaches, if only because the patient with aggressive drug-resistant disease may not remain eligible for or survive to receive sequential therapy. However, proof of superiority from administering multiple agents simultaneously rather than sequentially has been necessary to justify additive toxicity, a proof requiring randomized trials. Another challenge emerges from heterogeneity in the patient population as one patient’s molecular profile may be quite different from another with the same diagnosis. Or molecular results may suggest novel combinations that have not been studied. Nevertheless, such combinations may represent the best opportunity to defeat a particular cancer and constitute a “therapeutic imperative.”

This case report documents the utility of comprehensive genomic profiling to identify multiple synthetic lethal opportunities to design a novel therapy that targets the most vulnerable nodes in the tumor network, defined here as network-targeting combination therapy (NTCT). The idea of NTCT was first introduced nearly 20 years ago with the assertion that: “inhibiting activity of multiple nodes within the network can provide increased efficacy with potentially lower doses of each drug” (9). The patient achieved a definitive treatment benefit without toxicity, confirming, if only anecdotally, the potential efficacy of this approach.

## Case report

**Figure 1:**
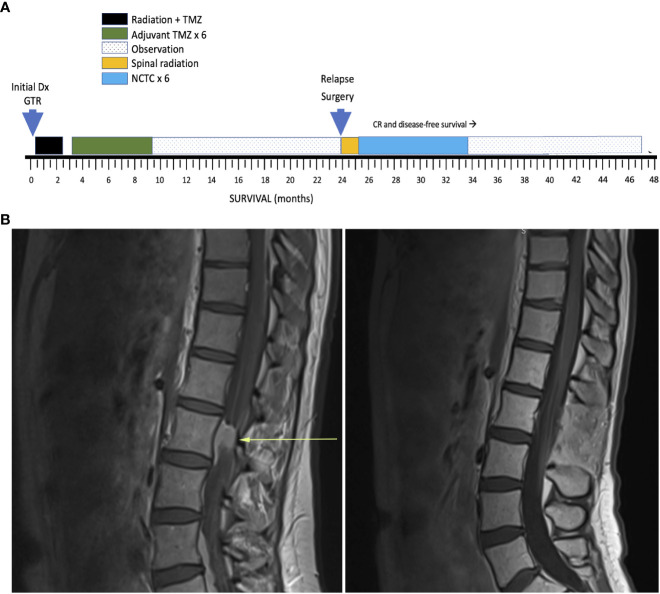
**(A)** Timeline of key events. GTR, Gross total resection; NTCT, network-targeting combination therapy (see text for details); CR, Complete remission. **(B)** Lumbar spine MRI T1 sagittal LEFT: at relapse and RIGHT: 10 months after NTCT during remission.

A 37-year-old woman with a germline DNA polymerase epsilon (*POLE)* mutation was diagnosed with right frontal glioblastoma, IDH wild type, with O^6^-guanine methyl transferase (*MGMT)* methylation (Timeline: **Figure 1**). Following gross total resection (GTR) and conventional chemoradiotherapy 60 Gy in 30 fractions with concurrent temozolomide (TMZ) (75mg/m^2^/day) followed by adjuvant (TMZ) (150 ➔ 200 mg/m^2^ x 5 days every 28 days) x 6 cycles, she achieved a disease-free survival of 24 months from diagnosis before developing back pain that led to the diagnosis of relapse with L3 spinal cord involvement (**Figure 1B**). The patient underwent laminectomy and GTR. Histologic sections of the resected tumor revealed invasion of the leptomeninges. Subsequently, her neurologic condition deteriorated with encephalopathy and she was diagnosed with disseminated LM-GBM with new MRI findings showing additional sites of LM disease. Because of the histologic diagnosis of LM invasion, CSF sampling was deemed unnecessary. Post-operative radiation therapy was administered to the L3 region of the spine along with high dose dexamethasone.

## Molecular diagnosis and theranosis

**Figure 2:**
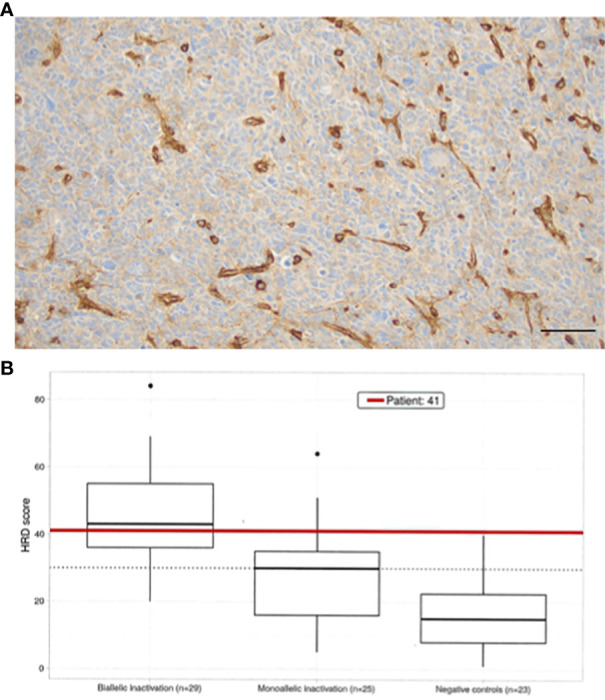
**(A)** HLA ABC immunostaining reveals complete loss in tumor cells, showing strong expression in tumor-associated vessels (X200, bar marks 50µ). **(B)** Homologous recombination deficiency scoring (10) using an open source method was employed (11). The HRD Score is calculated as the sum of all values for genomic instability, including scores for telomere-allelic imbalance (TAI), loss of heterozygosity (LOH), and large scale transition (LST). All three calculations result in independent unitless values of equal weight to determine the final HRD score for the patient’s sample. Boxplots of HRD score distribution in a cohort patients (N=30) reflects dependence with mutation status (control, mono-, bi-allelic inactivation) of one of the genes in the HR pathway: *ABRAXAS1, ARID1A, ATM, ATR, BAP1, BARD1, BLM, BRCA1, BRCA2, BRIP1, CHEK1, CHEK2, EMSY, FANCD2, FANCD2, FANCI, FANCM, MRE11, NBN, PALB2, RAD50, RAD51C, RAD51D, RECQL4, WRN*. ROC curve analysis (X-axis = sensitivity, negative controls HRD score <30 vs. Y-axis = specificity, positive samples with HRD >= 30) was employed to establish the threshold for HRD (=30) (CEGAT, Tubingen, DE).

We obtained MHC1 assessment by immunohistochemistry (IHC). Molecular profiling utilizing WES, homologous recombination repair deficiency (HRD) scoring, and microsatellite instability (MSI) testing was performed (**Table 1**). The relapsed tumor remained *MGMT*-methylated but acquired mismatch repair deficiency (MMRD) and MSI, mediated by *MSH2* and new *MSH6* loss of function (LOF) mutations, i.e., conferring total loss of MutSα function. Though the tumor was dramatically hypermutated with 275 mutations per megabase, the antigen presenting machinery (APM) was absent as assessed by IHC of MHC1 proteins which showed no membrane staining (**Figure 2A**). Despite the hypermutation in this cancer, the adaptive immune response is MHC1-dependent, making the benefit of PD-L1 checkpoint inhibitors contingent on antigen presentation machinery (APM) being intact. Given the impossibility of efficacy for PD-L1 blockade in this cancer, a decision was made not to pursue checkpoint immunotherapy.

**Table 1:** Mutations present in spinal relapse of GBM (furnished by the Center for Genomics and Transcriptomics (CEGAT; Tubingen, DE).

GENE	MUTATION	EFFECT	AF	IMPACT	SYNTHETC LETHAL
*POLE*	c.C>A; p.Asn363Lys	LOF	0.44	BER	N/A
*XRCC1*	c.G>A; p.395W	LOF	0.26		Olaparib
*MSH2*	c.G>T; p.Glu580*	LOF	0.50	MMR	Lomustine
	c.G>T; p.GLU647*	LOF	0.37		
*MSH6*	c.3261dup; p.Phe1088Leu fs*5	LOF	0.06		
	c.delG; p. Ala40Pro fs*41	LOF	0.13		
*EXO1*	p.R401*	LOF	0.30		
*BRCA1*	c.C>T; R24K	LOF	0.39	HRR	Olaparib
*BRIP1*	c.C>T; p.R581Q	LOF	24%		
*BRCA2*	c.C>T; p.Gln754*	LOF	0.31		
*PALB2*	c.dupT; p.Lys819*	LOF	0.44		
*ATM*	p.R1730*	LOF	0.20		
*TP53BP1*	c.G>A; p.P2S	LOF	20%	NHEJ	Olaparib
	c.G>A; p1721S	LOF	17%		
*PRKDC*	c.C>T; p.R2157H	LOF	56%		
	c.G>T; p.S360Y	LOF	74%		
	c.T>C; N1597S	LOF	94%		
*ERCC4*	c.C>T; p.P556L	LOF	36%	NER	Lomustine
*ATRX*	Splice variant	LOF	1.00	NHEJ, HRR Telomere Regulation	Lomustine, olaparib
*STK11*	c.delAGTA; p.?	LOF	0.39	REDOX Autophagy	Digoxin, Metformin, ascorbate
*TP53*	c.C>T; p.Arg213*	LOF	0.48	DNA Checkpoint NER	Lomustine
	c.C>T; p.Pro151Ser	SOF	0.70		

LOF, Loss of function mutation; SOF, Switch of function mutation; AF, allele fraction; BER, base excision repair; MMR, mismatch repair; HRR, Homologous recombination repair; NHEJ, non-homologous end joining repair.

**Figure 3:**
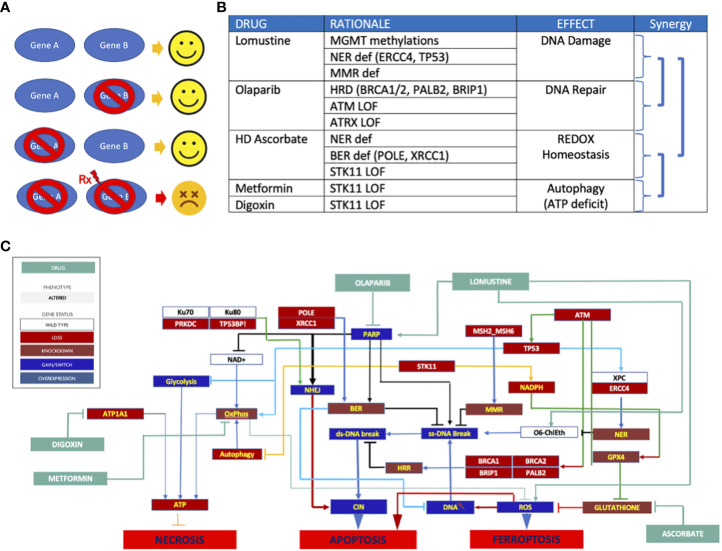
**(A)** Synthetic lethality principle. Gene A&B are partners in carrying out a vital function. Either one can be compromised with compromising cell survival. But when one partner is compromised and the other is targeted (Rx), the cell collapses. Normal cells without genomic abnormalities are not affected by Rx. **(B)** Synthetic lethal therapy rationale. Synergy emanates from 1) combining DNA damage with DNA repair targeting, 2) the use of REDOX homeostasis targeting to enhance DNA damage to trigger apoptosis without need for p53, 3) targeting ATP generation to decrease GPX4’s ability to enhance REDOX homeostasis, and 4) targeting ATP production to deprive the cell of energy needed for DNA repair; **(C)** Network targeting combination therapy. The schematic depicts the signaling pathway consequences of mutations and how these were exploited to trigger cell death.

A variety of synthetic lethal treatment options emerged from findings of homologous recombination repair (HRR) deficiency (**Figure 2B**), nucleotide excision repair deficiency, base excision repair (BER) deficiency, impaired REDOX homeostasis, and defective energy sensing. To capitalize the vulnerabilities caused by synthetic lethal relationships, the patient received an cocktail of lomustine, olaparib, digoxin, metformin, and high dose ascorbate (**Figure 3**). The mechanistic details of these synthetic lethal relationships and the rationale for therapy selection is discussed in **Supplemental File 1**.

## Implementing network targeting combination therapy

Because phase IB clinical trials of lomustine plus olaparib had never been pursued, extra care was exercised in the design of treatment. This challenge was addressed using the principle of *intra-patient dose escalation* (IDE), an innovative approach using sequential dose titration in a single patient (12, 13). IDE employed stepwise escalation of drug exposure in serial cycles of treatment until toxicity was encountered. Conventional dosing of lomustine (110 mg/m^2^ administered in 6-week cycles for 6 cycles) was administered. In addition, the patient received 3 days of olaparib 150 mg bid starting the day before lomustine. On the next cycle, 4 days of olaparib were used, and on the subsequent cycle, 5 days of olaparib were employed. The treatment was given with metformin 1,000 mg bid and digoxin 0.25 mg daily, along with intravenous high dose ascorbate 1g per kg biw. Serum digoxin levels were measured to ensure the drug remained within the desired range.

## Clinical course and outcome

The patient achieved a prompt complete remission. After escalation of olaparib to 5 days per chemotherapy cycle, a drop in nadir neutrophil and platelet counts (grade 2) was identified. No further dose escalation was attempted. The patient tolerated the novel combination uneventfully. No toxicity was encountered from utilizing metformin, digoxin, and intravenous high dose ascorbate. Olaparib and lomustine were discontinued after 6 cycles. With the use of MRI scanning at 3-month intervals and clinical assessment, she remains alive and disease free 23 months after diagnosis of relapsed LM-GBM and 47 months from initial diagnosis. She was last imaged at 21 months from relapse. She is free of neurologic deficits and returned to being a spouse, working full time, parenting a 5-year-old, and resumed running marathons.

## Discussion

Signaling pathway analysis of genomic aberrations depicts a complex dysregulated disease network mediating a correspondingly complex malignant phenotype. The difference between normal tissue and a cancer disease network is characterized by *key nodes* which are defined by oncogene addiction, synthetic lethal vulnerabilities arising from loss of tumor suppressor genes including DNA repair enzymes, and the master regulators of cell fate responsible for hallmark cancer behaviors. As such, comprehensive molecular diagnosis (CMD) reveals uniquely sensitive, disease-specific nodes which are typically involved with key cellular functions, including proliferation, survival, DNA repair, energy production, and REDOX homeostasis. Implicitly, targeting network nodes shared with normal tissue can be expected to cause dose-limiting toxicity. By comparison, targeting *disease-specific nodes* can lead to collapse of the cancer network with tolerable side effects. Synthetic lethal vulnerabilities do not exist in normal tissues unless a germline abnormality is present. Therefore, multiple cytocidal effects can be obtained in tumor tissue without causing significant harm to normal tissues. The absence of toxicity in this patient illustrates the favorable therapeutic index of targeting disease-specific synthetic lethalities. This approach represents quite a different treatment proposition than the conventional oncologic belief that toxicity is a prerequisite for treatment benefit.

In general, single agent approaches to glioblastoma have been either clinically futile or offered only transient disease control. Not surprisingly, complex networks are adept at maintaining homeostasis under stress and prone to robust adaptation and acquisition of resistance to single node targeting. However, some pathogenic mechanisms of cancer provide imperfect adaptation in the form of synthetic vulnerability. Optimal combination therapy can be guided by identifying these disease-specific nodes and strategically taking down *as many as possible* to fundamentally re-program the cell’s regulatory logic or deliver an irreparable insult that drives clinical efficacy and treatment benefit, conceived here as network-targeting combination therapy (NTCT). In this patient’s cancer, a combination of three DNA repair deficiencies together with compromised ATP production and oxidative stress resistance predicted synergistic efficacy for lomustine, olaparib, digoxin, metformin, and high dose ascorbate to trigger three discrete mechanisms of cell death: apoptosis, necroptosis, and ferroptosis.

Several objections may be offered. First a large, randomized trial testing the addition of the PARP inhibitor, veliparib, to TMZ in newly diagnosed GBM failed to enhance survival. However, a key weakness of that trial is that patients were not selected using a biomarker (14). In contrast to synthetic lethal targeting described here, the use of targeted therapy without a biomarker has not delivered a new drug for GBM patients in nearly two decades. While the non-targeted or general use of PARP inhibitors for GBM is not supported, HRD is a predictive biomarker that provides a substantial basis for PARP inhibitor deployment that has regulatory approval in four different malignancies.

Secondly, many oncologists dismiss drug re-purposing due to an evidence gap caused by a dearth of incentives for studying older drugs. However, in p53-deficient cancers that facilitate reversible senescence rather than apoptosis, the importance of inducing *p53-independent* forms of cell death such as ferroptosis should not be underestimated. Susceptibility to ferroptosis is recognized to play a key role in the outcome of GBM (15–23). Oxidative stress can also activate the intrinsic pathway of apoptosis, thus providing a crucial p53-independent trigger of cell death (24). Though we cannot measure the relative contribution of any component of the regimen that was employed, the *plurality* of strategies is a key determinant of successful network targeting.

Because of the relative rarity of patients with POLE-mutated and mismatch repair deficient disease, the survival of GBM patients with these genomic aberrations hardly has been studied. The few data available suggest that progression-free survival may be shorter and that overall survival may be longer in hypermutated cancers (25). However, there are too few patients to make a statistically confident assertion that hypermutated patients live longer. In any circumstance, patients with LM-GBM seldom survive more than 6 months, making it very unlikely that this patient’s clinical course can be ascribed to hypermutation.

While it may be tempting to dismiss this exceptional responder as an outlier, this patient’s success may be taken as an influential observation that addresses the challenge of designing meaningful combination therapy. Rather than the specific protocol that was selected, this report illustrates a *patient-centric* method of using comprehensive genomic analysis to derive a fundamental understanding of an individual patient’s unique cancer network that permits design of combination therapy that exploits the vulnerabilities within a unique cancer network.

Notably, the relapsed cancer had acquired mutations that were not present at initial diagnosis. While tumor evolution is usually thought to be a relentless process of increasing drug resistance and diminished treatability, this case shows that the acquisition of new genomic aberrations created vulnerable disease-specific nodes. As such, genomic instability led to drugs that would have been either unsuccessful or considerably less active as initial therapy. While genomic entropy is usually rewarded with “selection of the fittest,” as evidenced by the knockout of antigen presentation to facilitate immune evasion and TMZ resistance in this case, the haphazard nature of tumor evolution also created responsiveness to other untried drug strategies. The positive outcome of this case supports the utility of serial molecular profiling in the assessment of cancer at the time of disease progression. It also calls into question the practice of forecasting a limited survival without assessing for acquired changes in drug susceptibility.

The number of possible drug combinations is vastly greater than the number of phase IB clinical trials that could be conducted. To compound the problem, intellectual property laws and return on investment considerations rather than clinical merit are the prime determinants of study feasibility. Most combination regimens simply are not “investable” and a business case for studying many promising combinations will not emerge. Yet CMD of individual cancers is apt to reveal drug combinations which can address the goal of NTCT by attacking oncogenic drivers, synthetic lethal vulnerabilities, resistance mechanisms, redundant parallel pathways, and key convergence nodes, thus providing for the first time in history clear-sighted mechanistic insight how to take down a life-threatening disease. Thus, support for molecularly-based, novel combinations is needed.

Though there are few problems in oncology as grim as LM-GBM, comprehensive genomic diagnosis provided a design spec for novel NTCT that allowed us to identify and target multiple synthetic lethal opportunities resulting in an unprecedented clinical benefit. In summary, this case depicts how a rich *molecular portrait* can uncover otherwise hidden actionable intelligence and the possibility of personalized and highly effective combination therapy.

## Conclusion

NTCT introduces a translational methodology that addresses the unmet need of surpassing single agent therapy. Despite the poor prognosis associated with LM-GBM and relapsed GBM in general, effective therapy can be designed to target disease-specific nodes in the cancer’s complex adaptive and dysregulated network. In the era of commercially available multiomic data, comprehensive signaling pathway analysis provides an understanding of network nodes responsible for cancer’s most aggressive and lethal behaviors, but also its definitive vulnerabilities. As such, precision medicine has the potential to evolve beyond single mutation-single drug targeting to design personalized combinatorial strategies for individual patients with more ingenuity and payoff than molecularly blind drug development. With the patient’s partnership and consent, IDE represents an innovative method of executing the prescriptive program of delivering novel drug combinations in lieu of phase IB experience. While cases like the one presented here place increased responsibility on the care team administering novel treatment, there is no doubt that safety can be achieved with adequate research and caution. The use of NTCT to attack as many key nodes in the disease network as feasible demonstrates the utility of modeling the hallmarks of cancer biology in detail and inspires hope for defeating complex lethal malignancies. In so doing, we re-kindle the practice of the “art of medicine” by joining individual patient’s molecular results with the latest insights of science for much needed improvement of clinical outcomes.

## Patient perspective

When I learned about the recurrence in my spine including LMD, I knew we had to act quickly and decisively. Faced with this dire prognosis, my medical team and I were determined to find an effective treatment plan based on a thorough analysis of my tumor.

I am honored to have reached NED status thanks to the innovative treatment plan that led to a robust and complete response. This journey, which involved minimal side effects and was filled with hope, allowed me to regain my health and focus on my family and career once more.

NGS played a critical role in identifying targetable treatment options that would not have been considered under standard care protocols. I am amazed that this approach is not yet part of the standard treatment for all cancer patients.

With the knowledge I have about my tumor, I am filled with hope that I can proactively manage my health, maintain control of my journey, and explore additional treatment options if necessary. I am incredibly grateful for my medical team and the personalized approach they took, which has allowed me to be fully healthy again.

## Data availability statement

The original contributions presented in the study are included in the article/**Supplementary Material**. Further inquiries can be directed to the corresponding author.

## Ethics statement

Written informed consent was obtained from the individual(s) for the publication of any potentially identifiable images or data included in this article.

## Author contributions

The article was conceived and written by MC with additions by BS, SB, and NK. All authors contributed to the article and approved the submitted version.

## References

[1] Akmal S Ginalis EE Patel NV Aiken R Dicpinigaitis AJ Hanft SJ. Leptomeningeal disease in glioblastoma: endgame or opportunity? J Neurooncol (2021) 155:107–15. doi: 10.1007/s11060-021-03864-x 34623599

[2] Brennan CW Verhaak RG McKenna A Campos B Noushmehr H Salama SR. TCGA research network. The somatic genomic landscape of glioblastoma. Cell. (2013) 155(2):462–77. doi: 10.1016/j.cell.2013.09.034. Erratum in: Cell. 2014 Apr 24;157(3):753. PMC3910500 24120142

[3] Sakthikumar S Roy A Haseeb L Pettersson ME Sundstrom E Marinescu VD. Whole-genome sequencing of glioblastoma reveals enrichment of non-coding constraint mutations in known and novel genes. Genome Biol (2020) 21:127. doi: 10.1186/s13059-020-02035-x 32513296 PMC7281935

[4] Pećina-Šlaus N Kafka A Gotovac Jerčić K Logara M Bukovac A Bakarić R. Comparable genomic copy number aberrations differ across astrocytoma Malignancy grades. Int J Mol Sci (2019) 20(5):1251. doi: 10.3390/ijms20051251 30871102 PMC6429132

[5] Umehara T Arita H Yoshioka E Shofuda T Kanematsu D Kinoshita M. Distribution differences in prognostic copy number alteration profiles in IDH-wild-type glioblastoma cause survival discrepancies across cohorts. Acta Neuropathol Commun (2019) 7:99. doi: 10.1186/s40478-019-0749-8 31215469 PMC6580599

[6] Nawaz Z Patil V Thinagararjan S Rao SA Hegde AS Arivazhagan A. Impact of somatic copy number alterations on the glioblastoma miRNome: miR-4484 is a genomically deleted tumour suppressor. Mol Oncol (2017) 11:927–44. doi: 10.1002/1878-0261.12060 PMC5537698 28378523

[7] Bozdag S Li A Baysan M Fine HA. Master regulators, regulatory networks, and pathways of glioblastoma subtypes. Cancer Inform (2014) 13(Suppl 3):33–44. doi: 10.4137/CIN.S14027. Erratum in: Cancer Inform. 2014;13(Suppl 3):91. 25368508 PMC4214595

[8] Huang A Garraway LA Ashworth A Weber B. Synthetic lethality as an engine for cancer drug target discovery. Nat Rev Drug Discovery (2020) 19:23–38. doi: 10.1038/s41573-019-0046-z 31712683

[9] Araujo RP Doran C Liotta LA Petricoin EF. Network-targeted combination therapy: a new concept in cancer treatment. Drug Discovery Today: Ther Strategies (2004) 1(4):425–33. doi: 10.1016/j.ddstr.2004.11.004

[10] Sztupinszki Z Diossy M Krzystanek M Reiniger L Csabi I Favero F. Migrating the SNP array-based homologous recombination deficiency measures to next generation sequencing data of breast cancer. NPJ Breast Cancer (2018) 4(16):1–4. doi: 10.1038/s41523-018-0066-6 PMC6028448 29978035

[11] Available at: https://github.com/sztup/scarHRD#genomic-scar-scores.

[12] Medioni J Brizard M Elaidi R Reid PF Reid PF Benlhassan K. Innovative design for a phase 1 trial with intra-patient dose escalation: The Crotoxin study. Contemp Clin Trials Commun (2017) 7:186–8. doi: 10.1016/j.conctc.2017.07.008 PMC5898525 29696184

[13] van Tilburg CM Milde T Witt R Ecker J Hielscher T Seitz A. Phase I/II intra-patient dose escalation study of vorinostat in children with relapsed solid tumor, lymphoma, or leukemia. Clin Epigenet. (2019) 11:188. doi: 10.1186/s13148-019-0775-1 PMC6902473 31823832

[14] Sarkaria JN Ballman KV Kizilbash SH Sulman EP Giannini C Mashru SH. Randomized phase II/III trial of veliparib or placebo in combination with adjuvant temozolomide in newly diagnosed glioblastoma (GBM) patients with MGMT promoter hypermethylation (Alliance A071102). J Clin Oncol (2022) 40:16_suppl,2001–2001. doi: 10.1200/JCO.2022.40.16_suppl.2001

[15] Zhuo S He G Chen T Li X Liang Y We W. Emerging role of ferroptosis in glioblastoma: Therapeutic opportunities and challenges. Front Mol Biosci (2022) 9:974156. doi: 10.3389/fmolb.2022.974156. 17 August 2022 Sec. Molecular Diagnostics and Therapeutics. 36060242 PMC9428609

[16] Xiao D Zhou Y Wang X Zhao H Nie C Jiang X. A ferroptosis-related prognostic risk score model to predict clinical significance and immunogenic characteristics in glioblastoma multiforme. Oxid Med Cell Longev (2021) 2021:9107857. doi: 10.1155/2021/9107857 34804371 PMC8596022

[17] Dong J Zhao H Wang F Jin J Ji H Yan X. Ferroptosis-related gene contributes to immunity, stemness and predicts prognosis in glioblastoma multiforme. Front Neurol (2022) 13:829926. doi: 10.3389/fneur.2022.829926 35359663 PMC8960280

[18] Hu Z Mi Y Qian H Guo N Yan A Zhang Y. A potential mechanism of temozolomide resistance in glioma-ferroptosis. Front Oncol (2020) 10:897. doi: 10.3389/fonc.2020.00897 32656078 PMC7324762

[19] Zhuo S Chen Z Yang Y Zhang J Tang J Yang K. Clinical and biological significances of a ferroptosis-related gene signature in glioma. Front Oncol (2020) 10:590861. doi: 10.3389/fonc.2020.590861 33330074 PMC7718027

[20] Zhang X Jin S Shi X Liu S Li K Liu G. Modulation of tumor immune microenvironment and prognostic value of ferroptosis-related genes, and candidate target drugs in glioblastoma multiforme. Front Pharmacol (2022) 13:898679. doi: 10.3389/fphar.2022.898679 35571123 PMC9095828

[21] Tian Y Liu H Zhang C Liu W Wu T Yang X. Comprehensive analyses of ferroptosis-related alterations and their prognostic significance in glioblastoma. Front Mol Biosci (2022) 9:904098. doi: 10.3389/fmolb.2022.904098 35720126 PMC9204216

[22] Yang FC Wang C Zhu J Gai QJ Mao M He J. Inhibitory effects of temozolomide on glioma cells is sensitized by RSL3-induced ferroptosis but negatively correlated with expression of ferritin heavy chain 1 and ferritin light chain. Lab Invest (2022) 102:741–52. doi: 10.1038/s41374-022-00779-7\ 35351965

[23] Song Q Peng S Sun Z Heng X Zhu X. Temozolomide drives ferroptosis via a DMT1-dependent pathway in glioblastoma cells. Yonsei Med J (2021) 62(9):843–9. doi: 10.3349/ymj.2021.62.9.843 PMC8382730 34427071

[24] Cadenas E. Mitochondrial free radical production and cell signaling. Mol Aspects Med (2004) 25(1-2):17–26. doi: 10.1016/j.mam.2004.02.005 15051313

[25] Kim H Lim KY Park JW Kang J Won JK Lee K. Sporadic and Lynch syndrome-associated mismatch repair-deficient brain tumors. Lab Invest (2022) 102:160–71. doi: 10.1038/s41374-021-00694-3 PMC8784316 34848827

[26] Human cancer: from precurable to curable. Commemorating the 65th birthday of dr. James F. Holland. New york, may 29, 1990. Mt Sinai J Med (1992) 59(5):373–415. Available at: https://pubmed.ncbi.nlm.nih.gov/1359407/. 1359407

