# Supplementary material for: Network-targeting combination therapy of leptomeningeal glioblastoma using multiple synthetic lethal strategies: a case report
Source: Front Oncol. 2023 Oct 31;13:1210224. doi: 10.3389/fonc.2023.1210224 (PMC10644375; doi:10.3389/fonc.2023.1210224)
Supplement: Supplementary file 1 [file DataSheet_1.docx]

Supplemental File 1.

Rationale for olaparib, digoxin, metformin, and high dose ascorbate.

**Avoidance of Immunotherapy**

The cancer had no membrane staining of the MHC1, thus compromising antigen presentation, and obviating the possibility of checkpoint immunotherapy response utilizing PD-L1-PD1 antibodies. Biallelic mutations in *TP53* would be expected to compromise transcription of two key genes involved in antigen presentation: the endoplasmic reticulum (ER) aminopeptidase-1/2 (*ERAP1/2)* responsible for peptide processing; and transporter associated with antigen presentation-1/2 (*TAP1/2)* responsible for escorting the MHC1-peptide complex from the ER to the plasma membrane.^[^^[[1]](#endnote-1)]^  Additionally, mutations involving *HLA-A/C, ERAP2* and *TAP2* would directly compromise APM. Without intact MHC1 on the plasma membrane, the possibility of effective checkpoint immunotherapy was eliminated despite the extraordinarily high TMB,^[^^[[2]](#endnote-2)]^ a contributing cause why only 62% of hypermutated cancers respond to checkpoint immunotherapy.^[^^[[3]](#endnote-3)]^

**Selection of Cytotoxic Therapy**

Intact MMR is a prerequisite for TMZ efficacy^[^^[[4]](#endnote-4)]^ and thus, MMRD precluded a second course of TMZ. But compared to cancers with intact MMR, MMRD *enhances* response to lomustine.^[^^[[5]](#endnote-5),^^[[6]](#endnote-6),^^[[7]](#endnote-7)]^ Additionally, *TP53* loss handicaps the apoptotic response to TMZ leading to senescence rather than apoptosis.^[^^[[8]](#endnote-8)]^  On the other hand, *TP53* mutations also compromise nucleotide excision repair (NER) pathway by diminishing transcription of xeroderma pigmentosum, complementation group C (*XPC),* a key component of NER.^[^^[[9]](#endnote-9)]^ NER is the primary mediator reversing bulky side chain alkylation, including chloroethylation of O^6^-guanine caused by lomustine. As a result, *TP53* mutations confer sensitivity to lomustine.^[^^[[10]](#endnote-10)]^ MGMT also reverses alkylation caused by lomustine.^[^^[[11]](#endnote-11)]^  Hence the triumvirate of MMRD, *TP53* mutation, and *MGMT* silencing suggested lomustine would be highly effective based on three synthetic lethal mechanisms.

**Basis for PARP Inhibitor Selection**

In the absence of MGMT and intact NER, lomustine causes intrastrand cross-linking of DNA that generates double-stranded DNA breaks (DSB).^[^^[[12]](#endnote-12)]^ Normally, the homologous recombination repair (HRR) pathway promotes error-free repair of DSB. But in this case the presence of *BRCA1, BRIP1, BRCA2* and *PALB2* LOF mutations pointed to homologous recombination repair deficiency (HRD). Additionally, newly acquired deleterious mutations of ATM, a DNA checkpoint, compromised the activation of BRCA1 in response to double-strand-DNA breaks (DSB),^[^^[[13]](#endnote-13)]^ while *ATRX* loss also impaired HRR and generated responsiveness to poly-ADP ribose phosphorylase (PARP) inhibition.^[^^[[14]](#endnote-14)]^ In fact, formal HRD scoring of 41 was found, within the range of biallelic inactivation indicating marked compromise of HRD. (FIGURE 2B) Normally, PARP translocates to sites of DNA damage. In turn, PARP trapping by PARP inhibitors disables base excision repair (BER), thereby inducing single-stranded DNA breaks (SSB) that are converted to DSB by the replication machinery.^[^^[[15]](#endnote-15)]^  Subsequently, PARP inhibition results in enhanced DNA-PKcs (*PRKDC*) activity and upregulation of error-prone NHEJ to create an untenable level of chromosomal instability (CIN). As a result, HRD possesses synthetic lethality with PARP yielding high response rates to PARP inhibitors,^[^^[[16]](#endnote-16)]^  In this case, the utility of PARP inhibitor therapy is predicted by mutations affecting 6 synthetic lethal partners,^[^^[[17]](#endnote-17)]^ while synergy is predicted from the combination of the PARP inhibitor, olaparib, combined with the DNA damaging agent, lomustine. (FIGURE 3A/B)

Remarkably, *PRKDC* loss enhances sensitivity to PARP inhibition and lowers the IC50 of PARP inhibitors by 5 to 10x, creating another insight regarding synthetic lethality.^[^^[[18]](#endnote-18)]^ However, *TP53BP1* loss is a credentialed mechanism of PARP inhibitor resistance and was observed in a proportion of alleles.^[^^[[19]](#endnote-19)]^  This defect in NHEJ prompted concern whether PARP inhibition would be sufficient.

**Exploiting Defective REDOX Homeostasis**

Notably, the base excision repair (BER) system handles oxidative DNA damage. In this cancer, *POLE* and *XRCC1* mutations compromise BER and enhance sensitivity to oxidative stress. Because *STK11* normally facilitates REDOX homeostasis by producing NADPH, a co-factor for glutathione peroxidase and thioredoxins,^[^^[[20]](#endnote-20)]^ the *STK11* LOF mutation handicaps GPX4 for regenerating glutathione thus enhancing oxidative stress.^[^^[[21]](#endnote-21)]^ With this rationale, high dose ascorbate was adopted to enhance oxidative stress by consuming glutathione.^[^^[[22]](#endnote-22)]^ In addition to DNA damage, sufficient oxidative stress causes p53-independent collapse of mitochondrion outer membrane potential to trigger apoptosis (i.e., MOMP↑→ Δψ_m_↓ → OxPHOS↓ 🡪 Cytochrome C release 🡪 Caspase activation), a potentially crucial mechanism in cancers with an apoptotic handicap emanating from *TP53* knockout.^[30]^ Additionally, ROS cause lipid peroxidation to precipitate ferroptosis, i.e., a non-apoptotic form of cell death, providing another strategy to enhance the efficacy of lomustine. While the impact of chemotherapy on mitochondrial DNA enhances oxidative stress that accounts for a substantial portion of cytotoxicity as well as chemotherapy side effects of fatigue and neurocognitive disorders, enhanced sensitivity to oxidative stress caused by BER deficiency and *STK11* mutation in this cancer represent disease-specific vulnerabilities marking an opportunity for a clinically meaningful therapeutic index.

**Exploiting Defective Energetics**

Hyperactivation of PARP caused by DNA damage chemotherapy generates critical depletion of NAD+ resulting in loss of mitochondrial ATP production. In fact, activation of PARP1 by DNA damage causes depletion of 50%–80% of total cellular NAD^+^ leading to cell death.^[^^[[23]](#endnote-23)]^ Severe ATP deficiency precipitates programmed necrosis or necroptosis, representing another caspase-independent form of cell death.^[^^[[24]](#endnote-24)]^  Though *STK11* LOF mitigates the effectiveness of immunotherapy,^[^^[[25]](#endnote-25),^^[[26]](#endnote-26),^^[[27]](#endnote-27)]^ it also diminishes the cells capacity to respond to low ATP states by hampering activation of AMPK and autophagy.^[^^[[28]](#endnote-28)]^ In cell line experiments, ATP synthesis pathways involving Complex I of the electron transport chain and the Na^+^K^+^/ATPase membrane pump, *ATP1A1*, are both synthetic lethal partners of *STK11*.^[^^[[29]](#endnote-29),^^[[30]](#endnote-30),^^[[31]](#endnote-31)]^  Hence, this vulnerability was targeted with metformin (a complex 1 inhibitor) and digoxin (an ATP1A1 inhibitor) to deprive the cell of ATP needed for DNA repair, thus enhancing the efficacy of lomustine as well as precipitating necroptosis. (FIGURE 3C)

1. Leone P, Shin E-C, Perosa F, Vacca A, Dammacco F, Racanelli V. MHC Class I Antigen Processing and Presenting Machinery: Organization, Function, and Defects in Tumor Cells. *J Natl Cancer Institute* (2013) 105:1172–87. doi: 10.1093/jnci/djt184 [↑](#endnote-ref-1)
2. Dhatchinamoorthy K,  Colbert JD, and  Rock KL.^.^  Cancer Immune Evasion Through Loss of MHC Class I Antigen Presentation. *Front. Immunol*. 2021; 09: 636568. [doi.org/10.3389/fimmu.2021.636568](https://doi.org/10.3389/fimmu.2021.636568) [↑](#endnote-ref-2)
3. Le DT, Uram JN, Wang H, Bartlett BR, Kemberling H, Eyring AD, et al. PD-1 blockade in tumors with mismatch-repair deficiency. N Engl J Med. 2015; 372: 2509–2520.doi:10.1056/NEJMoa1500596 [↑](#endnote-ref-3)
4. D'Atri S, Tentori L, Lacal PM, Graziani G, Pagani E, Benincasa E, et al. Involvement of the mismatch repair system in temozolomide-induced apoptosis. *Mol Pharmacol*. 1998; 54(2):334-41. DOI: [10.1124/mol.54.2.334](https://doi.org/10.1124/mol.54.2.334) [↑](#endnote-ref-4)
5. Aquilina G, Ceccotti S, Martinelli S, Hampson R, & Bignami M. N-(2-chloroethyl)-N'-cyclohexyl-N-nitrosourea sensitivity in mismatch repair-defective human cells. *Cancer Res*. 1998; 1;58(1):135-41. PMID: 9426069. [↑](#endnote-ref-5)
6. Aquilina G, Ceccotti S, Martinelli S, Soddu S, Crescenzi M, Branch P,  et al. Mismatch repair and p53 independently affect sensitivity to N-(2-chloroethyl)-N'-cyclohexyl-N-nitrosourea. *Clin Cancer Res*. 2000; 6(2):671-80. PMID: 10690553 [↑](#endnote-ref-6)
7. Fiumicino, S., Martinelli, S., Colussi, C., Aquilina , Leonetti C, Crescenzi M,  et al. Sensitivity to DNA cross-linking chemotherapeutic agents in mismatch repair-defective cells *in vitro* and in xenografts. *Int. J. Cancer*, 2000; 85: 590-596. DOI: [10.1002/(sici)1097-0215(20000215)85:4<590::aid-ijc23>3.0.co;2-o](https://doi.org/10.1002/(sici)1097-0215(20000215)85:4%3C590::aid-ijc23%3E3.0.co;2-o) [↑](#endnote-ref-7)
8. Mijit M, Caracciolo V, Melillo A, Amicarelli F, Giordano A. Role of p53 in the Regulation of Cellular Senescence. Biomolecules. 2020 Mar 8;10(3):420. doi: 10.3390/biom10030420. PMID: 32182711; PMCID: PMC7175209. [↑](#endnote-ref-8)
9. Adimoolam S, James M. Ford JM. p53 and DNA damage-inducible expression of the xeroderma pigmentosum group C gene. *Proceedings of the National Academy of Sciences*. 2002; 99(20): 12985-12990. DOI: [10.1073/pnas.202485699](https://doi.org/10.1073/pnas.202485699) [↑](#endnote-ref-9)
10. Nikolova T, Roos WP, Krämer OH, Strik HM, Kaina B. Chloroethylating nitrosoureas in cancer therapy: DNA damage, repair and cell death signaling. *Biochimica et Biophysica Acta (BBA) - Reviews on Cancer.* 2017;1868(1), 29-39. doi.org/10.1016/j.bbcan.2017.01.004. [↑](#endnote-ref-10)
11. Ludlum DB. The chloroethylnitrosoureas: sensitivity and resistance to cancer chemotherapy at the molecular level. Cancer Invest. 1997;15(6):588-98. doi: 10.3109/07357909709047601. PMID: 9412665. DOI: [10.3109/07357909709047601](https://doi.org/10.3109/07357909709047601) [↑](#endnote-ref-11)
12. Chakkath T, Lavergne S, Fan TM, Bunick D, Dirikolu L. Alkylation and Carbamylation Effects of Lomustine and Its Major Metabolites and MGMT Expression in Canine Cells. Vet Sci. 2015 Apr 24;2(2):52-68. doi: 10.3390/vetsci2020052. [↑](#endnote-ref-12)
13. Aglipay JA, Martin SA, Tawara H, Lee SW, Ouchi T. ATM Activation by Ionizing Radiation Requires BRCA1-associated BAAT1. *Journal of Biological Chemistry*, 281,14:9710-9718, ISSN 0021-9258, 2006. DOI: [10.1074/jbc.M510332200](https://doi.org/10.1074/jbc.m510332200) [↑](#endnote-ref-13)
14. Garbarino J, Eckroate J, Ranjini K., Jensen RB,  Bindra RS. Loss of ATRX confers DNA repair defects and PARP inhibitor sensitivity. *Translational Oncology*, 2021; 14( 9):101147. DOI: [10.1016/j.tranon.2021.101147](https://doi.org/10.1016/j.tranon.2021.101147) [↑](#endnote-ref-14)
15. Patel AG, Sarkaria JN, Kaufmann SH. Nonhomologous end joining drives poly(ADP-ribose) polymerase (PARP) inhibitor lethality in homologous recombination-deficient cells. Proc Natl Acad Sci U S A. 2011 Feb 22;108(8):3406-11. doi: 10.1073/pnas.1013715108. [↑](#endnote-ref-15)
16. Telli, M.L., Stover, D.G., Loi, S. *et al.* Homologous recombination deficiency and host anti-tumor immunity in triple-negative breast cancer. *Breast Cancer Res Treat* **171**, 21–31 (2018). <https://doi.org/10.1007/s10549-018-4807-x> [↑](#endnote-ref-16)
17. Paulet L, Trecourt A, Leary A,  Peron J, Descotes F, Mojgan Devouassoux-Shisheboran M, et al. Cracking the homologous recombination deficiency code: how to identify responders to PARP inhibitors. *European Journal of Cancer*. 2022; 166:87-99. [↑](#endnote-ref-17)
18. Gout J, Perkhofer L, Morawe M*, et al*. Synergistic targeting and resistance to PARP inhibition in DNA damage repair-deficient pancreatic cancer. *Gut*2021;70:743-760. [↑](#endnote-ref-18)
19. Nacson J, Krais JJ, Bernhardy AJ, Clausen E, Feng W, Wang Y, et al. BRCA1 Mutation-Specific Responses to 53BP1 Loss-Induced Homologous Recombination and PARP Inhibitor Resistance. Cell Rep. 2018 Sep 25;24(13):3513-3527.e7. doi: 10.1016/j.celrep.2018.08.086. [↑](#endnote-ref-19)
20. Jeon SM, Chandel NS, Hay N. AMPK regulates NADPH homeostasis to promote tumour cell survival during energy stress. *Nature*. 2012; 485(7400):661-5.  DOI: [10.1038/nature11066](https://doi.org/10.1038/nature11066) [↑](#endnote-ref-20)
21. Bonanno L, Zulato E, Pavan A, Attili I, Pasello G,  Conte PF, et al. LKB1 and Tumor Metabolism: The Interplay of Immune and Angiogenic Microenvironment in Lung Cancer. *Int J Mol Sci*. 2019; 20(8):1874. DOI: [10.3390/ijms20081874](https://doi.org/10.3390/ijms20081874) [↑](#endnote-ref-21)
22. Renner O, Burkard M, Michels H, Vollbracht C, Sinnberg T, Venturelli S. Parenteral high‑dose ascorbate - A possible approach for the treatment of glioblastoma (Review). *Int J Oncol. 2021;* 58: 35. DOI: [10.3892/ijo.2021.5215](https://doi.org/10.3892/ijo.2021.5215) [↑](#endnote-ref-22)
23. Hurtado-Bagès S, Knobloch G, Ladurner AG, Buschbeck M. The taming of PARP1 and its impact on NAD^+^ metabolism. Mol Metab. 2020 Aug;38:100950. doi: 10.1016/j.molmet.2020.01.014. Epub 2020 Feb 12. PMID: 32199820; PMCID: PMC7300387. [↑](#endnote-ref-23)
24. Morales J, Li L, Fattah FJ, Dong Y, Bey EA, Patel M, Gao J, Boothman DA. Review of poly (ADP-ribose) polymerase (PARP) mechanisms of action and rationale for targeting in cancer and other diseases. Crit Rev Eukaryot Gene Expr. 2014;24(1):15-28. doi: 10.1615/critreveukaryotgeneexpr.2013006875. [↑](#endnote-ref-24)
25. Della Corte CM, Byers LA. Evading the STING: LKB1 Loss Leads to STING Silencing and Immune Escape in KRAS-Mutant Lung Cancers. Cancer Discov. 2019 Jan;9(1):16-18. doi: 10.1158/2159-8290.CD-18-1286. PMID: 30626603; PMCID: PMC8330553. [↑](#endnote-ref-25)
26. Eria Eksioglu, Gabriela M. Wright, Trent R. Percy, Kenneth L. Wright, W. Douglas Cress. Loss of CX3CL1 expression mediates immune evasion in *STK11* mutated lung adenocarcinomas. [abstract]. In: Proceedings of the American Association for Cancer Research Annual Meeting 2023; Part 1 (Regular and Invited Abstracts); 2023 Apr 14-19; Orlando, FL. Philadelphia (PA): AACR; Cancer Res 2023;83(7_Suppl):Abstract nr 4454. [↑](#endnote-ref-26)
27. Koyama S, Akbay EA, Li YY, Aref AR, Skoulidis F, Herter-Sprie GS, et al. STK11/LKB1 Deficiency Promotes Neutrophil Recruitment and Proinflammatory Cytokine Production to Suppress T-cell Activity in the Lung Tumor Microenvironment. Cancer Res. 2016 Mar 1;76(5):999-1008. doi: 10.1158/0008-5472.CAN-15-1439. Epub 2016 Feb 1. PMID: 26833127; PMCID: PMC4775354. [↑](#endnote-ref-27)
28. Pons-Tostivint E, Lugat A, Fontenau JF, Denis MG, Bennouna J. *STK11/LKB1* Modulation of the Immune Response in Lung Cancer: From Biology to Therapeutic Impact. *Cells*. 2021; 10(11):3129. doi: 10.3390/cells10113129.  [↑](#endnote-ref-28)
29. Massimo Moro M, Caiola E, , Ganzinelli M, Zulato E, Rulli E, Marabese M,  et al. Metformin Enhances Cisplatin-Induced Apoptosis and Prevents Resistance to Cisplatin in Co-mutated KRAS/LKB1 NSCLC. *Journal of Thoracic Oncology.* 2018; 13(11): 1692-1704. doi: 10.1016/j.jtho.2018.07.102.

    [↑](#endnote-ref-29)
30. Kim, N., Yim, H., He, N. Lee CJ, Kim JH, Choi JS, *et al.* Cardiac glycosides display selective efficacy for STK11 mutant lung cancer. *Sci Rep.* 2016; 6, 29721. doi: 10.1038/srep29721.  [↑](#endnote-ref-30)
31. Ndembe G, Intini I, Perin E, Marabese M, Caiola E, Mendogni P, et al. LKB1: Can We Target an Hidden Target? Focus on NSCLC. *Frontiers in Oncology*. 2022; 12:889826. doi: 10.3389/fonc.2022.889826.  [↑](#endnote-ref-31)
